# Rhinology and Laryngology

**Published:** 1897-05

**Authors:** 


### SELECTIONS AND ABSTRACTS.

### RHINOLOGY AND LARYNGOLOGY.

EDITED BY L. B. GRANDY, M.D.

### SPURS OF THE NASAL SEPTUM AS FACTORS IN DISEASES OF THE RESPIRATORY TRACT.

Dr. E. F. Parker, of Charleston, calls attention to the radical difference, etiologically as well as clinically, between spurs and deflections of the septum. The former are direct results of hypertrophic or vaso-motor changes along the sutural lines, causing perichondritis or hyperostosis, and perhaps secondarily producing stenosis, while the latter are primarily of traumatic origin, hypertrophic changes being only superinduced when the deviation is sufficient to cause stenosis.

Slight deviations produce no practical interference with nasal respiration, and demand no attention, as a rule; but spurs of a size apparently too small to impair the respiratory functions of the nasal fossæ frequently affect other parts by a reflex action. In actual practice the symptoms arising from these two morbid conditions may often be the same, when originating solely from mechanical obstruction to nasal respiration; yet in those cases where the patient is aware of no impediment to normal breathing slight deviations will produce no discomfort, while small spurs may cause extra-nasal symptoms. These observations have often been confirmed by the beneficial results which follow the removal of seemingly insignificant spurs in patients who were unaware of any stenosis.

Spurs along the sutural lines dividing the perpendicular plate of the ethmoid and vomer more commonly give rise to reflex symptoms than those situated along the suture separating the triangular cartilage and hard palate, because the former interfere with the region of the middle turbinate membrane, which has its sensitiveness or irritability increased by the distribution of the filaments of the olfactory nerve.

Septal spurs, by reason of their situation, exert a powerful influence on the whole respiratory apparatus. The peculiar nasal twang, the so-called "dead voice," nasal catarrh, or hypertrophic rhinitis, chronic post-nasal pharyngitis, purulent rhinitis, and chronic pharyngitis, are all familiar and common results of nasal stenosis and interference with nasal respiration due to septal spurs.

They are important factors in the persistence of adenoid growths, somewhat rarely found in adults, the atrophy of the latter at puberty being prevented by the irritation and vascularity induced by the spurs. Their influence on catarrhal and sclerosing inflammation of the middle ear is somewhat doubtful, as the obstruction occurs upon the unaffected side very frequently.

Epistaxis, which is a common symptom of septal spurs, may lead to a suspicion of pulmonary hemorrhage, as in a case reported. The effect of septal growths on the larynx, trachea and bronchi were discussed, and cases of dysphonia, aphonia, bronchitis, dyspnea, cough, dysphagia, cervical neuralgia, and paroxysmal retching and hawking, were cited or reported as sometimes of intra-nasal origin.—*The Laryngoscope.*

---

### CLEANLINESS IN CATARRH.

Dr. Edwin Pynchon, in an article in the *Annals of Ophthalmology*, calls attention to the widely varying formulæ of Dobell's Solution given by different authors, and incidentally mentions what is a really practical question in the treatment of naso-pharyngeal catarrh.

Numerous preparations are widely advertised as adapted for cleansing purposes in the nasal cavity, and are possibly of real merit, but the price asked for the product is so exorbitant, that to people of moderate means the expense is a serious factor, while to the poor, it is beyond their purse, and in each case, after the prescription has, perhaps, been filled once, they cease its use, and go back to the home remedy of salt and water of varying strength, and usually with disastrous results.

The Seiler's tablets, made by different manufacturers, also vary in strength and composition, and our experience has taught us that se-veral of those on the market cannot be used without causing g.eat smarting, and even pain.

The fluid used in cleansing the nasal cavities, in both atrophic and hypertrophic rhinitis, should be of about the specific gravity of the serum of the blood, and this is acquired in the solution advised by Dr. Pynchon, which is as follows:

| | | |
|---|---|---|
| ℞ | Sodæ bicar | 2 ounces. |
| | Sodæ biborat | 2 ounces. |
| | Listerine (Lambert's) | 8 ounces. |
| | Glycerine | 1½ pints. |

One ounce of this formula added to a pint of water, yields a bland and pleasant alkaline solution with a specific gravity of 1.015.

The addition of the listerine takes the place of the carbolic acid in the original formula, and is a decided advantage, as it imparts a pleasant taste, and is quite as efficacious as the acid.

The common use of listerine and water should be superseded by the addition of the alkaline solution given, and in the preparation thus made, we have all the advantages of any cleansing agent, and it can be furnished at a price commensurate with all pockets.—*Atlantic Medical Weekly.*

---

### EUCAINE IN LARYNGOLOGY AND RHINOLOGY.

Dr. Joseph S. Gibb, of the Philadelphia Polyclinic, contributes a comprehensive report under above title to the college organ (January 23, 1897), from which we quote a few extracts:

A 2 per cent. solution has been found entirely serviceable. . . The effect of solutions in this strength became manifest in two to three minutes and lasted fully fifteen to twenty minutes. As with cocaine, there is a very great difference in individuals as to length of time in producing and the duration of anesthesia. In a very few it was difficult to detect any anesthetic effect. . . In nineteen cases, in which these experiments were employed, no apparent difference in the contractile effects of the two drugs (eucaine and cocaine) could be detected in fifteen; in three cases cocaine was decidedly more effective; and in one the difference seemed to be in favor of the eucaine.

A record of eight cases is then quoted, and the author concludes:

That it has a powerful anesthetic influence upon mucous surfaces is indisputable. Not the slightest evidence has been observed in a single case of a poisonous action of the drug, and, therefore, we are justified in asserting that it is safer in this respect than cocaine. Positive statements, however, should not be made until we have had longer experience in its use.—*American Therapist.*

